# Mitochondrial Nucleic Acid as a Driver of Pathogenic Type I Interferon Induction in Mendelian Disease

**DOI:** 10.3389/fimmu.2021.729763

**Published:** 2021-08-26

**Authors:** Alice Lepelley, Timothy Wai, Yanick J. Crow

**Affiliations:** ^1^Université de Paris, Imagine Institute, Laboratory of Neurogenetics and Neuroinflammation, Inserm UMR 1163, Paris, France; ^2^Mitochondrial Biology Group, Institut Pasteur CNRS UMR 3691, Paris, France; ^3^Medical Research Council Human Genetics Unit, Institute of Genetics and Cancer, University of Edinburgh, Edinburgh, United Kingdom

**Keywords:** type I interferonopathy, mitochondrial disease, type I interferon, autoinflammation, mitochondria, mtDNA, mtRNA, innate immunity

## Abstract

The immune response to viral infection involves the recognition of pathogen-derived nucleic acids by intracellular sensors, leading to type I interferon (IFN), and downstream IFN-stimulated gene, induction. Ineffective discrimination of self from non-self nucleic acid can lead to autoinflammation, a phenomenon implicated in an increasing number of disease states, and well highlighted by the group of rare genetic disorders referred to as the type I interferonopathies. To understand the pathogenesis of these monogenic disorders, and polyfactorial diseases associated with pathogenic IFN upregulation, such as systemic lupus erythematosus and dermatomyositis, it is important to define the self-derived nucleic acid species responsible for such abnormal IFN induction. Recently, attention has focused on mitochondria as a novel source of immunogenic self nucleic acid. Best appreciated for their function in oxidative phosphorylation, metabolism and apoptosis, mitochondria are double membrane-bound organelles that represent vestigial bacteria in the cytosol of eukaryotic cells, containing their own DNA and RNA enclosed within the inner mitochondrial membrane. There is increasing recognition that a loss of mitochondrial integrity and compartmentalization can allow the release of mitochondrial nucleic acid into the cytosol, leading to IFN induction. Here, we provide recent insights into the potential of mitochondrial-derived DNA and RNA to drive IFN production in Mendelian disease. Specifically, we summarize current understanding of how nucleic acids are detected as foreign when released into the cytosol, and then consider the findings implicating mitochondrial nucleic acid in type I interferonopathy disease states. Finally, we discuss the potential for IFN-driven pathology in primary mitochondrial disorders.

## Introduction

Most cells are equipped with cytosolic sensors involved in the intracellular surveillance of pathogens, leading to the rapid induction of an antiviral IFN response (1). DNA is recognized by cyclic GMP–AMP synthase (cGAS), activating endoplasmic reticulum (ER)-resident Stimulator of interferon genes (STING) (2). STING then traffics to the Golgi, eventually inducing the transcription of IFN. Along similar lines, RNA species are recognized by RIG-I-like receptors (RLRs), RIG-I (retinoic acid-inducible gene I) and MDA5 (melanoma differentiation-associated protein 5), activating the adaptor protein mitochondrial antiviral-signaling protein (MAVS) on mitochondria, again leading to IFN induction (1).

The type I interferonopathies are rare genetic diseases characterized by chronic upregulation of type I IFN signaling (3). Strikingly, the majority of type I interferonopathy-related disease genes identified to date encode molecules playing a role in nucleic acid processing or sensing, highlighting the importance of active mechanisms to prevent antiviral responses triggered by self nucleic acids, and the challenge of self/non-self discrimination (4). Indeed, aberrant sensing of self nucleic acids has been increasingly implicated in a diversity of pathologies including autoimmunity, genome instability syndromes, cancer, neurodegeneration and senescence (4).

To better understand pathogenesis, it is important to determine the source of the self nucleic acids detected by innate (antiviral) sensors. Recent studies have established that genomic DNA represents such an agonist when abnormally exposed to cGAS (2, 5–7). Interestingly, in some type I interferonopathies, and in senescence, DNA and RNA derived from endogenous retroelement expression may also represent ‘self’-derived nucleic acid capable of triggering IFN signaling (8–12). Notably, beyond the nucleus, mitochondria constitute the other intracellular source of self nucleic acids, possessing their own DNA (mtDNA) and RNA (mtRNA) enclosed by the mitochondrial membranes (13). The mtDNA encodes 13 respiratory chain proteins, 22 tRNAs and 2 rRNAs, with the remaining ∼1,300 mitochondrial proteins imported after translation from the nuclear genome (13). Since mitochondria constitute the cytosolic remnants of the endosymbiosis of proteobacteria within eukaryotic cells (14), mtDNA and mtRNA demonstrate immunostimulatory characteristics of pathogens, with the potential to be misinterpreted as foreign. Thus, each cell contains hundreds to thousands of copies of circular double stranded (ds) mtDNA molecules, which are hypomethylated, devoid of histones, exposed to reactive oxygen species (ROS) and poorly repaired. Furthermore, bidirectional transcription generates long dsRNA and uncapped mRNAs, and mtDNA transcription and replication give rise to single stranded DNA, RNA-DNA hybrids and G-quadruplexes (13, 15, 16), all of which have immunostimulatory capacity. Indeed, there is increasing recognition of the potential of mitochondrial-derived nucleic acids (mtNA) to act as agonists of the IFN signaling machinery, possibly contributing to complex autoinflammatory diseases such as systemic lupus erythematosus (SLE) (17), as well as neurodegeneration (18, 19) and cancer (20, 21).

In this review we focus on mtNA cytosolic sensing leading to IFN induction. However, we note that mtDNA and other mitochondrial molecules [cardiolipins, formyl peptides, mitochondrial ROS (mROS)] can also trigger other innate sensing pathways. For example, these molecules can engage the inflammasome, resulting in interleukin 1β (IL1β)-mediated inflammation (22–25) and endosomal Toll-like receptor 9 (TLR9) activation, leading to IFN and NF-κB-dependent inflammatory cytokine induction (26, 27). These pathways, also implicated in inflammatory diseases, are specific to discrete cell types and have been extensively reviewed elsewhere (15, 24, 25, 28, 29). mtDNA can also be released into the extracellular space, acting as a plasmatic marker and driver of systemic inflammation in autoimmunity, traumatic injury, lung inflammation and cardiovascular disease (30, 31).

## Mitochondrial Nucleic Acid Is Interferonogenic in the Cytosol

**Figure 1:**
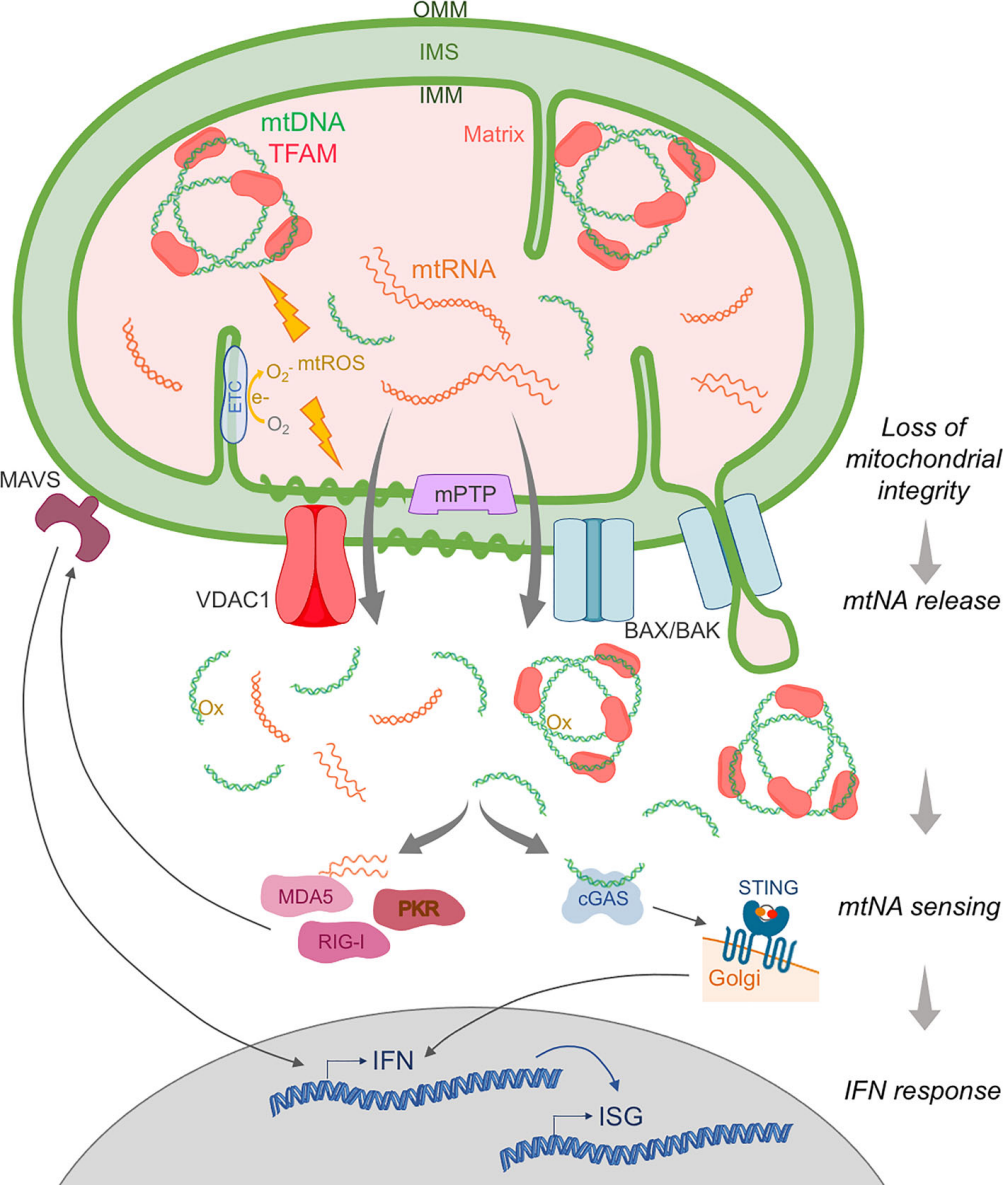
Main pathways of mitochondrial nucleic acid release and sensing. Upon extrinsic or intrinsic insult, the mitochondrial membrane integrity is compromised and mtDNA and mtRNA, normally contained within the double membrane, can be released into the cytosol. Release of mtDNA packaged into nucleoids by TFAM is mediated by outer mitochondrial membrane (OMM) perforation by BAX/BAK macropores, while mtDNA fragments devoid of TFAM are thought to egress through VDAC1 pores. Inner mitochondrial membrane (IMM) permeabilization to mtNA can involve herniation into BAX/BAK pores, destabilization due to oxidative stress (e.g. mitochondrial ROS (mtROS) generated from electrons (e_) leaking from the electron transport chain (ETC)), and/or opening of the mitochondrial permeability transition pore (mPTP). In the cytosol, mtDNA, oxidized (Ox) mtDNA and mtRNA are detected as foreign by innate cytosolic sensors of immunostimulatory DNA (e.g. cGAS), and RNA (e.g. RIG-I, MDA5, PKR). These receptors then activate the adaptor molecules STING and MAVS, respectively, leading to the induction of IFN and subsequent IFN-stimulated gene (ISG) expression. IMS, inner membrane space.

mtNA remains ‘immunologically inert’ when retained inside the two nested compartments delimited by the mitochondrial membranes: the matrix enclosed in the inner mitochondrial membrane (IMM), and the inner membrane space (IMS) between the IMM and the outer mitochondrial membrane (OMM) (25) (**Figure 1**). Thus, an increasing number of reports indicate that loss of mitochondrial integrity and compartmentalization, as a result of mitochondrial stress, can allow the release of mtNA, and subsequent interaction with cytosolic receptors leading to IFN induction (25, 28).

Seminal studies first described IFN induction due to mtDNA release upon abortive apoptosis in 2014 (32, 33), the process of which was detailed in real-time by high-resolution imaging in 2018 (34, 35). Upon mitochondrial apoptosis triggered by the activation of BAX (Bcl-2 associated-X protein) and BAK (BCL-2 homologous antagonist/killer), BAX/BAK pores are formed in the OMM, releasing proapoptotic factors from the IMS, and leading to activation of caspases 9, 3 and 7 and apoptotic cell death (36). However, when BAX/BAK activation is induced together with caspase inhibition, mtDNA complexed to TFAM into nucleoids is released into the cytosol, sensed by cGAS-STING, and IFN induced (32, 33), suggesting a role for programmed cell death completion in preventing inflammatory mtNA sensing. Despite these insights, the question of how mtDNA might first cross the IMM remained. A contribution of the mitochondrial permeability transition pore (mPTP), an IMM channel that allows for non-selective diffusion of low molecular weight solutes and water (<1.5 kDa) (37), as well as mROS-dependent destabilization of the IMM, have been suggested to facilitate such egress (23, 30, 38, 39). Recent work has also highlighted IMM herniation, through BAX/BAK macropores, followed by IMM permeabilization independent of mPTP opening (34, 35) (**Figure 1**). However, these mechanisms of IMM crossing are difficult to reconcile with the size of mtDNA nucleoids (40).

Remarkably, an increasing number of situations associated with mitochondrial stress have been linked to the release and sensing of mtNA, mostly mtDNA through cGAS-STING, and the induction of IFN (28). These include environmental insults, oxidative stress, ‘suboptimal’ mitochondrial function, mitochondrial dysfunction due to mutations (mitochondrial disease detailed below), proteotoxic stress, and infection (24, 25, 30). Such stress, typically evidenced by impaired oxidative phosphorylation and ATP production, metabolic imbalance, loss of mitochondrial potential and mROS induction, results in a loss of mitochondrial integrity and release of mitochondrial components. Notably, this phenomenon can be considered ‘physiological’ when induced by pathogens, promoting an antiviral state (41–45). Cytosolic mtDNA then constitutes a second messenger, initiating the antiviral response. Along similar lines, upon genotoxic stress, mtNA release is sensed as a sign of genomic instability and can activate DNA repair pathways (20, 46, 47). Interestingly, in the context of SLE, mitochondrial oxidative stress enhances the interferonogenic potential of mtDNA itself by oxidation (17, 48, 49) (**Figure 1**). Indeed, mitochondrial hyperpolarization causes slippage of electrons onto molecular oxygen, which is reversible by treatment with the antioxidant N-acetylcysteine *in vitro* and *in vivo*; also demonstrating therapeutic efficacy in patients with SLE (50, 51).

Studies of mtNA release upon different mitochondrial stresses have both reinforced the role of BAX/BAK macropores (21, 43, 46, 52, 53) and mPTP (17, 54), and elucidated further relevant mechanisms and the type of mtDNA species egressing. As an example, upon loss of mitochondrial endonuclease EndoG, oxidative stress triggers voltage-dependent anion-selective channel 1 (VDAC1) oligomerization and the formation of pores in the OMM, with subsequent release into the cytosol of mtDNA fragments, rather than TFAM-bound nucleoids, a situation relevant to SLE (17), viral infection (55) and altered mitochondrial proteostasis (56) (**Figure 1**). In some instances, a combination of VDAC1 pores in the OMM, and mPTP for IMM permeabilization, facilitates complete mitochondrial envelope opening (17, 19). In the case of infection, viroporins and other microbial proteins have been proposed to perforate mitochondrial membranes (43, 57). Host inflammasome effector Gasdermin D can also permeabilize mitochondria (58), and the RLR adaptor MAVS has been described as a mitochondrial membrane remodeler (43, 59, 60), although the release of mtNA remains to be observed in this situation.

Analogous to the processes involving mtDNA described above, a few studies have reported mtRNA relocalization leading to innate immune stimulation. Upon loss of p53, mtRNA is sensed by cytosolic MDA5 and RIG-I (61), possibly related to the opening of the mPTP (62). In a model of Huntington’s disease, mtRNA accumulated in the cytosol, activating the RNA sensor protein kinase R (PKR) and subsequent IFN-stimulated gene (ISG) expression (63). Interestingly, mtDNA ds breaks can lead to mtRNA sensing by RIG-I in the cytosol (46). Additionally, mtRNA sensing might be relevant in the context of TLR7-dependent IFN induction upon ablation of the mitophagy actor IRGM1 in mouse macrophages (TLR7 being an RNA sensor) (64), and in the RNA sensing/MAVS-dependent mtDNA cytosolic leakage upon influenza virus M2 viroporin expression (43).

Although speculative, loss of membrane integrity might also allow the entry of nucleic acid sensors into mitochondria. Indeed, mitochondrial membranes contain complexes involved in the import of nuclear-encoded mitochondrial proteins (65), enabling, for example, aberrant entry of mutated TDP-43 in the context of amyotrophic lateral sclerosis (ALS) (19). This phenomenon could explain the observation of a basal interaction between PKR and mitochondrial dsRNA (66). Interestingly, although cGAS recruitment to IMM hernias, for mtDNA sensing, is not observed during abortive apoptosis (35), cGAS contains a cryptic mitochondrial targeting sequence, and truncated cGAS can translocate to mitochondria and become activated (67).

Mitophagy constitutes the selective degradation of damaged mitochondria by autophagy, participating in mitochondrial quality control (25). *A priori*, removal of dysfunctional mitochondria leaking mtNA may act as a safeguard against detrimental IFN induction. Thus, although not directly implicated in mtNA retention, mitophagy activation could limit mitochondrial immunogenicity and pathogenicity (68). As such, autophagy and mitophagy have been shown to dampen the innate immune response induced by mtNA leakage and sensing (17, 21, 23, 27, 69, 70), and, even, to be triggered by pathogens themselves (71, 72). Consistently, defective mitophagy can enhance sensing (18, 73) [reviewed in (24, 25, 74)]. Importantly, such ‘mitophagic maintenance’ has been suggested to have pathogenic relevance in autoimmune diseases [e.g. Sjögren’s syndrome (64) and SLE (75)], and Parkinson’s disease (18).

Summarizing, our understanding of how mitochondrial stress and damage leads to mitochondrial rupture and IFN-inducing mtNA release has recently broadened. However, these data have been mostly derived from *in vitro* studies, mouse models, or through biomarker correlations (28–30).

## Immunostimulatory Mitochondrial Nucleic Acid in Type I Interferonopathies

**Figure 2:**
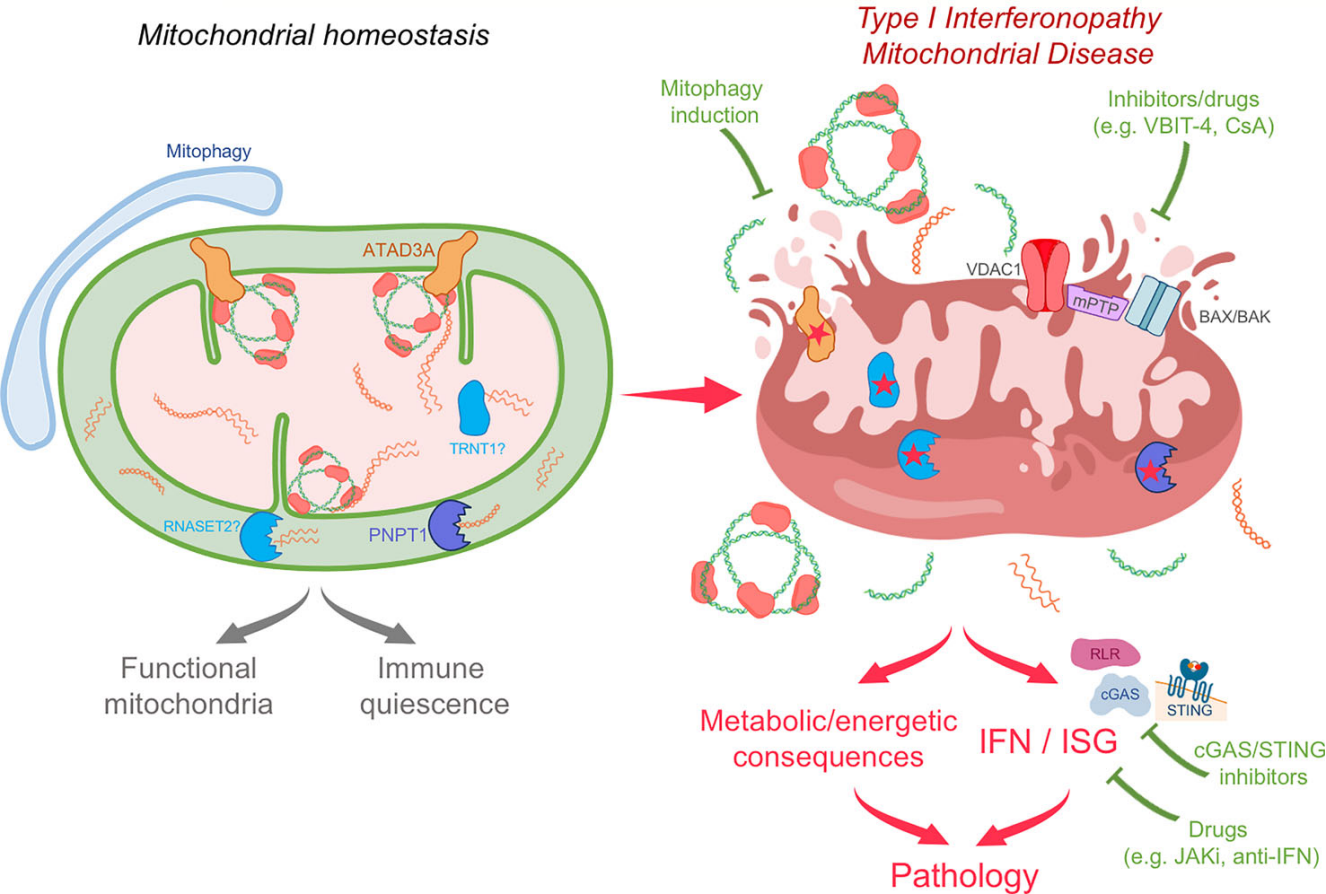
Potentially common pathogenic mechanisms in type I interferonopathy and mitochondrial disease and therapeutic perspectives. Due to the immunostimulatory potential of mtNA, active processes are required to ensure mitochondrial homeostasis and immunological quiescence. These include degradation of mtdsRNA by PNPT1, maintenance of mtDNA and mitochondrial structure by molecules such as ATAD3A, and metabolism of mtRNA by proteins such as RNASET2 and TRNT1. More general mitochondrial quality control mechanisms are involved as well, including mitophagy and mitochondrial proteases. Defects in these processes may result in both mitochondrial disease and a type I interferonopathy state. Indeed, loss of function of PNPT1 and ATAD3A, and possibly of RNASET2 and TRNT1, can lead to mitochondrial stress and mtNA cytosolic leakage and sensing. In the type I interferonopathies linked to mutations in PNPT1 and ATAD3A, in addition to mitochondrial dysfunction, there is chronic induction of IFN and ISGs. This might also be a feature of other mitochondrial diseases, which may have gone undetected due to lack of relevant investigations. Identifying immunostimulatory mtNA and IFN induction as pathogenic mechanisms opens new therapeutic perspectives, including by inhibition of type I IFN signaling (JAK inhibitors (JAKi), anti-IFN system antibodies), mtNA sensing (cGAS/STING inhibitors), mitochondrial membrane opening [VBIT-4, cyclosporin A (CsA)], or by mitophagy induction.

Providing strong evidence of the potential of mtNA to induce pathological IFN induction in humans, inappropriate sensing of mtNA has recently been demonstrated in Mendelian diseases due to mutations in *PNPT1*, *NGLY1* and *ATAD3A* (73, 76, 77) (**Figure 2**). These observations highlight mechanisms involved in mitochondrial homeostasis directly relevant to the avoidance of aberrant sensing of mtNA in human health.

Dhir et al. described loss of the mtRNA exoribonuclease PNPT1 to result in an accumulation and cytosolic leakage of dsRNA derived from bidirectional mtDNA transcription, triggering IFN through a BAX/BAK-dependent mechanism (76). Consistent with the type I interferonopathy disease spectrum, patients carrying hypomorphic mutations in *PNPT1* display enhanced IFN signaling in blood (and, in some cases, intracerebral calcification, a well-known clinical feature of IFN activation) (76, 78). Mutations in *NGLY1*, encoding conserved deglycosylation enzyme NGLY1, cause a severe neurodevelopmental phenotype (79, 80). In a mouse model, Yang et al. showed that loss of NGLY1 also results in chronic activation of cytosolic nucleic acid sensing pathways, likely induced by a combination of mtRNA and mtDNA (73). Here, mitochondrial quality control may be the broad link between NGLY1 and mtNA homeostasis, involving mitophagy and/or proteasome function. Relating to the clinical phenotype, an apparent resistance to viral infection was noted, and increased ISG expression recorded in patient-derived cell lines, although the contribution of IFN to the observed neuropathology remains to be defined (73).

Very recently, we directly implicated, for the first time, mtDNA sensing in IFN induction in a Mendelian disease context (77). Specifically, we identified two patients demonstrating chronically enhanced IFN signaling in blood and features of systemic sclerosis, a rare autoimmune disorder where IFN signaling and mtDNA have been suggested to play a role in pathogenesis (81–83). Surprisingly, these patients carried dominant negative heterozygous mutations in *ATAD3A*, encoding the mitochondrial AAA ATPase protein ATAD3A, previously described to cause mitochondrial disease with neurological features (84, 85). Importantly, we also observed enhanced IFN signaling in patients with a predominant neurological clinical phenotype, suggesting a consistent link between *ATAD3A* mutations and IFN signaling. We demonstrated cytosolic leakage of mtDNA, and cGAS-STING-dependent IFN induction (**Figure 2**). Although VDAC1 oligomers seemed to be relevant, the mechanisms of mtDNA cytosolic relocalization will require further study, and a direct role for ATAD3A cannot be excluded. Indeed, ATAD3A has been implicated in multiple mitochondrial processes, including mtDNA maintenance, mitochondrial ultrastructural organization, mitochondria-ER junction stabilization and cholesterol biosynthesis (86–90).

Interestingly, hypomorphic mutations in *RNASET2* and *TRNT1* may also be associated with perturbed mtNA homeostasis leading to enhanced IFN signaling (91, 92). Mutations in *RNASET2* cause a phenotype mimicking congenital viral infection, reminiscent of some type I interferonopathies (78, 93), and RNASET2 has been suggested to play a role in mitochondrial ribosomal RNA degradation in the IMS (94). Further, IFN pathway induction has been observed in some patients with mutations in *TRNT1* (91), and TRNT1 is required for tRNA aminoacylation of mitochondrial and cytosolic tRNA, with protein dysfunction leading to defective mitochondrial translation (95). Of interest, a Mendelian metabolic disease due to deficiency in mevalonate kinase, involved in the biosynthesis of cholesterol and isoprenoids, may also involve mitochondrial damage, mtDNA release and sensing (96). However, to date, only inflammasome pathway activation, leading to IL1β induction, has been implicated mechanistically (97). Since anti-IL1β signaling treatments are only partially effective in this context (98, 99), one might speculate that enhanced IFN signaling may be contributive to the phenotype (100–102). We also note that in iPSC-derived motor neurons from patients carrying TDP-43 mutations associated with ALS, mtDNA release and sensing lead to IFN induction, although the relevance of IFN signaling to ALS remains unclear (19).

Interestingly, mtRNA relocalization and sensing have been only infrequently implicated in the numerous studies reporting *in vitro* mitochondrial stress leading to IFN induction (46, 61, 63, 64). However, given that mtDNA depletion, used *in vitro* to prove the implication of mtDNA, also results in mtRNA depletion, mtRNA may have a currently unappreciated role in this context (even when demonstrating DNA-dependent sensing). Further, since the majority of dsRNA detected in the cytosol is of mitochondrial origin (28), and PKR binds mtRNA at steady state (66), constitutive leakage of mtRNA may prevent the recording of acute mtRNA sensing. Indeed, mtRNA may be more ‘mobile’ than mtDNA, since it is untethered to the mitochondrial membrane (unlike mtDNA nucleoids organized around TFAM) (13). Alternatively, it may be that mtRNA abundance and containment are tightly regulated, with redundant mitochondrial and cytosolic nucleases preventing their accumulation (103, 104), and/or that mtRNA cytosolic leakage and sensing are more harmful to cells *in vitro*, leading to toxic translational arrest through activation of the PKR pathway (66, 105).

The above cases illustrate the potential of the study of type I IFN-related Mendelian disease to define novel cellular functions, revealed by hypomorphic or gain-of-function mutations, providing insights into poorly understood mechanisms of mtNA retention.

## An Overlap Between Type I Interferonopathy and Mitochondrial Disease?

Mutations in more than 350 nuclear or mtDNA-encoded genes are known to result in mitochondrial disease, involving diverse tissues and responsible for heterogenous clinical phenotypes (106, 107). Clinical characterization is lengthy and difficult, and, where a genetic diagnosis is unavailable, relies on the identification of metabolic changes, neuropathological manifestations and mitochondrial dysfunction in muscle biopsy (106, 108, 109). While the contribution of defective oxidative phosphorylation and bioenergetic and metabolic stress is clear, the findings summarized above suggest that mtNA sensing driving IFN signaling may also be relevant to mitochondrial disease pathology. Thus, disease caused by mutations in *PNPT1* and *ATAD3A* are considered as bona fide primary mitochondrial disorders, and might now also be included in the type I interferonopathy grouping (76, 77, 106). Mitochondrial disease is typically accompanied by various types of mitochondrial dysfunction and/or due to specific defects in mtNA metabolism (106, 108), with the potential to cause mtNA release and sensing. Interestingly, it has been suggested that enhanced IFN signaling, linked to mtDNA cytosolic release, can occur in mitochondrial syndromes due to single large-scale mtDNA deletions, associated with clinical features overlapping with those seen in certain type I interferonopathies (such as basal ganglia calcification and skin lesions) (110). Additionally, some mitochondrial disease mouse models manifest exacerbated IFN signaling associated with engagement of cytosolic mtDNA sensing, e.g. upon loss of the mitochondrial proteases CLPP and YMEL1 (56, 111). Similarly, multisystemic dysfunction caused by mtDNA mutation accumulation in the proofreading-deficient POLG mutator mouse can be rescued by ablation of cGAS-STING activity or IFN signaling (112). Whether maladaptive inflammation is observed in the corresponding human mitochondrial diseases has not been explored.

It is important to emphasize that the evaluation of IFN signaling is still not routine in medical practice (113, 114), even for inflammatory diseases. Thus, even if autoinflammation is not typically reported in mitochondrial disease (115–117), we suggest that increased IFN signaling may be more broadly associated with mitochondrial dysfunction than is currently appreciated, potentially contributing to the clinical phenotype beyond bioenergetic or metabolic defects. Indeed, enhanced interferon signaling related to ATAD3A dysfunction was only recognized six years after gene mutations were initially described (77, 84, 85). Further indication of a possible relationship between mitochondrial disease and the type I interferonopathies comes from shared clinical features, such as intracranial calcification being an established sign in both settings (3, 118). Similarly, bilateral striatal necrosis is recurrent in mitochondrial disease, and consistently described in the context of mutations in both *PNPT1* (78) and the type I interferonopathy due to ADAR1 loss-of-function (119). Likewise, dystonia, peripheral neuropathy, hypertrophic cardiomyopathy and isolated spastic paraparesis, recorded in patients with mutations in *ATAD3A* (77, 84, 85), are features of interferon-related disease (120, 121).

## Perspectives

The power of studying Mendelian diseases lies in the deconvolution of complex processes relevant to human health. Thus, if further validated, an overlap between type I IFN-related and mitochondrial diseases would, in combination, facilitate our understanding of the safeguards in place to prevent inappropriate mtNA sensing leading to harmful IFN induction. *In vitro* screening approaches using knock down strategies are hampered by the potential induction of cellular toxicity, and do not necessarily afford the mechanistic insights that studying gain-of-function and hypomorphic mutations can provide. Indeed, the diversity of processes described so far as contributing to mtNA leakage and sensing upon mitochondrial stress, suggests that the immunological quiescence of mtNA is achieved through currently incompletely understood, and difficult to predict, active processes.

Clearly, the extent and significance of an overlap between mitochondrial dysfunction and type I IFN induction in human disease needs to be defined, perhaps foremost by the systematic screening of IFN signaling status in the blood and cerebrospinal fluid of mitochondrial disease patients. Such studies could have important clinical implications, both from a diagnostic and therapeutic perspective. Thus, therapies targeting IFN signaling, and showing clinical benefits in type I interferonopathies, are available (JAK inhibition) (122, 123), and others are in development [e.g. anti-IFN (receptors) antibodies and STING inhibitors] (28, 124, 125) (**Figure 2**). These could provide a new therapeutic angle for mitochondrial disorders, most lacking real treatment options (126). Proving the contribution of pathogenic IFN signaling to disease will require the observation of clinical improvement with such IFN-targeted therapies. Therapeutic approaches could also target broader processes beyond blocking IFN signaling in diseases implicating mtNA sensing, e.g. through the removal of ruptured mitochondria by inducing mitophagy (17, 74, 127, 128) (**Figure 2**). In this regard, two patients with mutations in *ATAD3A* have been treated for inflammatory features by rapamycin (77), used as an immunosuppressant (129), but which, one could speculate, may act as a mitophagy inducer in this case. Indeed, rapamycin has shown benefit in a few patients with a mitochondrial encephalopathy (130), and a clinical trial of rapamycin is planned for the mitochondrial disorder Leigh syndrome (126).

Taking account of the potential sensing of escaped mtNA in mitochondrial disease might shed light on pathogenesis, and explain poorly understood features of these diseases such as variable clinical penetrance, specificity of tissue involvement only partially correlated to bioenergetic demands, and exacerbation of mitochondrial disease after infection or metabolic challenge (106, 109). Conversely, mitochondrial damage due to mutations in mitochondrial genes as a cause of type I interferonopathies lacking a genetic cause is also worthy of closer consideration.

## Author Contributions

AL wrote the first draft of the manuscript and designed figures. TW and YC provided valuable comments and edited the manuscript. All authors contributed to the article and approved the submitted version.

## Funding

AL is supported by funding from the European Union’s Horizon 2020 research and innovation program under the Marie Skłodowska-Curie grant agreement No. 892311. YC acknowledges that work relating to this manuscript has received funding from the European Research Council (ERC) under the European Union’s Horizon 2020 research and innovation programme (grant agreement No 786142) and State funding from the Agence Nationale de la Recherche under “Investissements d’avenir” program (ANR-10-IAHU-01). TW acknowledges funding from the ERC (grant agreement No. 714472).

## Conflict of Interest

The authors declare that the research was conducted in the absence of any commercial or financial relationships that could be construed as a potential conflict of interest.

## Publisher’s Note

All claims expressed in this article are solely those of the authors and do not necessarily represent those of their affiliated organizations, or those of the publisher, the editors and the reviewers. Any product that may be evaluated in this article, or claim that may be made by its manufacturer, is not guaranteed or endorsed by the publisher.
